# The metagenome of *Caracolus marginella* gut microbiome using culture independent approaches and shotgun sequencing

**DOI:** 10.1016/j.dib.2017.11.043

**Published:** 2017-11-22

**Authors:** Robert J. Rabelo-Fernandez, Kevin Santiago-Morales, Luis Morales-Vale, Carlos Rios-Velazquez

**Affiliations:** Biology Department, University of Puerto Rico at Mayagüez, Puerto Rico

## Abstract

Studies underestimate the microbial diversity and genotypic traits in the snails’ microbiome. *Caracolus marginella,* a land snail native to Caribbean islands, can adapt to different environments. Our research focused on the generation of a metagenomic library from *C. marginella* gut, to further explore the diversity and functional traits. Thirty specimens of *C. marginella* were collected from the four regions of Puerto Rico. High molecular weight (40 kb) metagenomic libraries were generated using a direct DNA isolation method. DNA was end-repaired and ligated into a pCCFOS1 fosmid vector; then, the cloned DNA was transduced into *Escherichia coli* EPI300. The master pool library contains approximately 60,200 clones and restriction enzyme digestion showed that 90% of the library contains insert. After removing the fosmid and host genome sequences, 567,015 sequences were analyzed using the MG-RAST online server. The Bacteria domain was the most abundant (82.15%), followed by viruses (16.49%), eukaryotes (0.83%) and archaea (0.31%). The *Proteobacteria* (51.47%) was predominant in the gut environment, followed by unidentified virus (16.28%), and *Actinobacteria* (8.52%). *Escherichia coli*, *Streptomyces avermitilis*, and *Burkholderia* sp. were the most abundant species present. Subsystem functional analysis showed that 35.00% of genes belong to transposable elements, 10.00% of genes belong to clustering-based subsystems, 4.00% of genes belong to the production of cofactors and secondary metabolites, and 2.00% resistance to antibiotics and toxic compounds. The data generated in this research is the first metagenomic examination of a snail gut in Puerto Rico, and will serve as a baseline to start understanding of *C. marginella* gut microbiome.

**Specifications Table**

**Table:** 

Subject area	Biology
More specific subject area	Diversity and Functional structure of the gut microbiome of the snail *Caracolus marginella*
Type of data	Text Files and Figures
How data was acquired	Shotgun Sequencing: MiSeq (Illumina), MR DNA Laboratories, USA.
Data format	Raw and Analyzed
Experimental factors	Gut dissection and washed, Metagenomic Library Construction, Shotgun Sequencing and Analysis.
Experimental features	Metagenomic library was constructed and shotgun sequencing were performed from sample obtained from the *Caracolus marginella* gut.
Data source location	The specimens were collected in Puerto Rico at the followed locations: Carolina (18°17′37.75′ lat; -65°55′5.19′ lng), Canóvanas (18°19′59.73′ lat; −65°53′20.53′ lng), Guayama (17°58′46.58′ lat; −66°6′10.44′ lng), Mayagüez (18°12′87.61′ lat; −67°7′36.0′ lng) and Camuy (17°58′37.75′ lat; −66°55′20.4′ lng).
Data accessibility	Generated metagenomic data was submitted into NCBI database under the accession number: SAMN07420447

**Value of the data**

• This project present the microbial diversity and functional profile of *Caracolus marginella* gut microbiome by using metagenomics and shotgun sequencing.
• This project generated the first snail gut metagenomic libraries in Puerto Rico.
• These data are the first steps in gaining a better understanding of the *Caracolus marginella* gut microbiome. Also, the data obtained will provide insights in the diversity, biomedical, and biotechnological potential in the microflora of terrestrial invertebrates in Puerto Rico.

## 1 Data

**Fig. 1:**
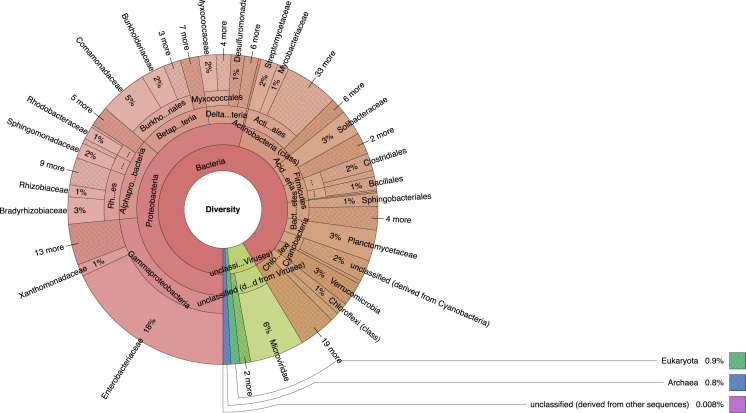
Community structure of the snail *Caracolus marginella* gut metagenome. The taxonomic analysis reveals that Bacteria domain was the most abundant (82.15%), followed by viruses (16.49%), eukaryotes (0.83%), archaea (0.31%), other sequences (0.13%) and unidentified sequences (0.09%). The metagenome was constituted of a total of 50 microbial phyla from which the most abundant was *Proteobacteria* (51.47%), followed by unclassified derived from virus (16.28%), *Actinobacteria* (8.52%), *Bacteroidetes* (3.83%) and *Plactomycetes* (3.54%). The data represents the combination of the DNA extracted from *C. marginella* gut from the four regions of Puerto Rico.

**Fig. 2:**
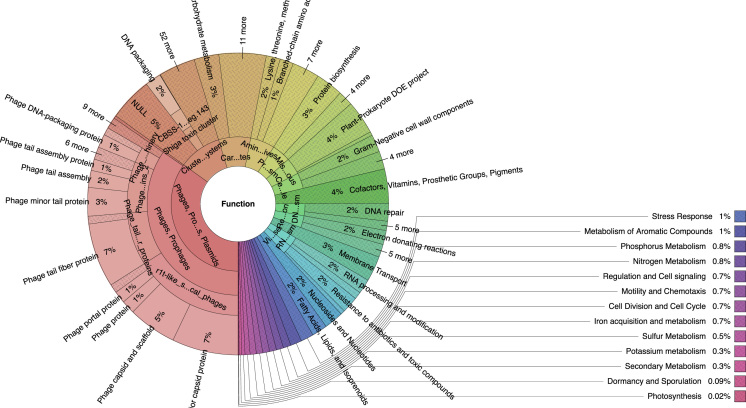
Functional structure of the snail *Caracolus marginella* gut metagenome using subsystem annotation. Subsystem functional examination showed that 35.00% of genes belong to transposable elements (phages, prophages, and plasmids), 10.00% of genes belong to clustering-based subsystems, 4.00% of genes belong to the production of cofactors and secondary metabolites (vitamins, prosthetic groups and pigments). In addition, genes belonging to metabolism of carbohydrates (8.00%), amino acids (6.00%), proteins (5.00%), DNA metabolism (4.00%), cell wall (4.00%), miscellaneous (4.00%), membrane transport (3.00%) and resistance to antibiotics and toxic compounds (2.00%) are also mentioned. The data represents the combination (pooling) of the DNA extracted from *C. marginella* gut from four distant regions of Puerto Rico.

In the past years, the study of microbiomes has been focus of the metagenomics, since its role in metabolic processes of a host is essential for the host's survival. Microbiome is defined as the collective genomes of the microbes (composed of bacteria, archaea, bacteriophage, fungi, protozoa and viruses) that live inside and on the body of one organism [7]. The microbes confer metabolic capabilities such as protection against pathogens, education of the immune system, and influence directly or indirectly most of the physiologic functions. Culture dependent approach allows to access 0.1–1% of the organisms in most habitats [2]. Culture independent approach such as, metagenomics not only intends to explore the rest of 99% of the population, which remains unnoticed, but the rest of cultivable part. This technique can access the genes and gene products present in the environment allowing their study, understanding, and manipulation. In this order, metagenomic library was constructed from *C. marginella* and shotgun sequencing were performed. Fig. 1 showed the community structure of the snail *Caracolus marginella* gut metagenome. Fig. 2 provide and overview of the functional structure of the snail *Caracolus marginella* gut metagenome using subsystem annotation.

## 2 Experimental design, materials, and methods

### 2.1 Sample collection

Thirty *Caracolus marginella* snails were collected by hand during the years 2013 to 2015. In order, to have a representation from different gut microbiomes from *C. marginella* adapted to different environments of Puerto Rico, five different locations from four regions in the Island were established and used as collection sites. The places where specimens were collected are Carolina (18°17′37.75′ lat; −65°55′5.19′ lng), Canóvanas (18°19′59.73′ lat; -65°53′20.53′ lng), Guayama (17°58′46.58′ lat; -66°6′10.44′ lng), Mayagüez (18°12′87.61′ lat; −67°7′36.0′ lng) and Camuy (17°58′37.75′ lat; −66°55′20.4′ lng). The coordinates for each specimen location were determined using a global position system (GPS).

### 2.2 Metagenomic library generation

In order, to extract the gut from specimens the snail was dissected according to Akpan's method [1]. The shell broke up from snail body using a sterile tweezers and scissors. Following, the gut was removed from the animal body using knives and tweezers. These instruments must be different to those used in the previous step. Once the gut was removed out of the body, the gut cavity was washed carefully with physiological water (a 1–10 μl pipette was necessary in the process) according to Valle et al. [6]. This procedure maintains the sample integrity and prevents the removal of the gut epithelial cells. This physiological water used to wash the gut became our sample, from where DNA extraction was performed.

Samples from physiological water used to wash snail gut, was filtrated through sterile cheesecloth to remove tissue and debris. The samples were filtered through a 0.45 µm membrane (MF-Millipore™) to trap the cells in the filter. DNA extraction using Metagenomic DNA Isolation Kit for water (Epicentre, USA) were performed to the membranes following the manufacturer's specifications. Extracted DNA was end-repaired and ligated into the fosmid vector pCCFOS1. Recombinant DNA was packaged and transduced to *Escherichia coli* Epi300 *via* T5 bacteriophages using CopyControl™ Fosmid Library Production Kit (Epicentre, USA) using the manufacturer's specifications. After plating the library, the total number of clones in the four metagenomic libraries was determined. Random clones were selected to confirm the presence of pCCFOS1 vector and insert environmental DNA by restriction enzyme digestion using *Bam*H1 (New England Biolabs).

### 2.3 Metagenome sequencing and pCCFOS1 vector removal

MetaDNA was extracted and purified using Midiprep kit (QIAGEN, USA) following manufacturer's specifications. DNA was sent to MR DNA (http://www.mrdnalab.com) where the genomic library was generated using Nextera DNA Sample Preparation Kit (Illumina) and Qubit® dsDNA HS Assay Kit (Life Technologies). After DNA samples were measured (50 ng) and diluted (to 2.5 ng/μL), fragmentation and addition of adapter sequences was done. The library was diluted (to 12 pM) and sequenced using a six hundred cycle v3 Reagent Kit (Illumina) on the MiSeq (Illumina). The quality check was performed using FastQC [3]. We used the tools from the FastX toolkit to trim the sequences to Phred scores higher than 30, eliminate the sequencing adapters and convert the FastQ files to Fasta [4]. SeqClean (The Gene Indices Sequences Cleaning and Validation Script) [5] was used to remove the pCC1FOS vector from the metagenomic library sequences using a minimum identity of 65% and 10 bases as the minimum to be considered a hit. The genomic sequence of the bacterial host *E. coli* EPI300 T1R was extracted using as a base the genome of *E. coli* DH10B, which is the strain from were EPI300 was produced.

### 2.4 Taxonomic and functional insights

To generate a taxonomic profile and a functional *in silico* description of the samples, the metagenomic sequencing data was examined with the Rapid Annotation using Subsystems Technology for metagenomes (MG-RAST) online server.
